# Exposure to anticholinergic and sedative medication is associated with impaired functioning in older people with vertigo, dizziness and balance disorders—Results from the longitudinal multicenter study MobilE-TRA

**DOI:** 10.3389/fphar.2023.1136757

**Published:** 2023-03-03

**Authors:** Benedict Katzenberger, Daniela Koller, Ralf Strobl, Rebecca Kisch, Linda Sanftenberg, Karen Voigt, Eva Grill

**Affiliations:** ^1^ Institute for Medical Information Processing, Biometry, and Epidemiology, Faculty of Medicine, Ludwig Maximilian University of Munich, Munich, Germany; ^2^ Pettenkofer School of Public Health, Munich, Germany; ^3^ Munich Center of Health Sciences, Ludwig Maximilian University of Munich, Munich, Germany; ^4^ German Center for Vertigo and Balance Disorders, University Hospital Munich, Munich, Germany; ^5^ Institute of General Practice and Family Medicine, University Hospital Munich, Munich, Germany; ^6^ Department of General Practice/Medical Clinic III, Faculty of Medicine, University Hospital Carl Gustav Carus, Dresden, Germany

**Keywords:** vertigo, dizziness, balance disorders, anticholinergic and sedative medications, drug burden index, functioning

## Abstract

**Introduction:** Anticholinergic and sedative medication is prescribed for various conditions in older patients. While the general association between anticholinergic and sedative medication and impaired functioning is well established, its specific role in older individuals with vertigo, dizziness, and balance disorders (VDB) is still incompletely understood. The objective of this study was to investigate, whether an exposure to anticholinergic and sedative medication is associated with lower generic and lower vertigo-specific functioning in older patients with VDB.

**Methods:** Data originates from the longitudinal multicenter study MobilE-TRA with two follow-ups, conducted from 2017 to 2019 in two German federal states. Exposure to anticholinergic and sedative medication was quantified using the drug burden index (DBI). Generic functioning was assessed by the Health Assessment Questionnaire Disability Index, appraising the amount of difficulties in performing activities of daily living (ADL). Vertigo-specific functioning was measured using the Vestibular Activities and Participation (VAP) questionnaire, assessing patient-reported functioning regarding activities of daily living that are difficult to perform because of their propensity to provoke VDB (Scale 1) as well as immediate consequences of VDB on activities and participation related to mobility (Scale 2). Longitudinal linear mixed models were applied to assess the association of exposure to anticholinergic and sedative medication at baseline and the level of generic and vertigo-specific functioning status over time.

**Results:** An overall of 19 (7 from Bavaria) primary care physicians (mean age = 54 years, 29% female) recruited 158 (59% from Bavaria) patients with VDB (median age = 78 years, 70% female). Anticholinergic and sedative medication at baseline was present in 56 (35%) patients. An exposure to anticholinergic and sedative medication at baseline was significantly associated with lower generic functioning [Beta = 0.40, 95%-CI (0.18; 0.61)] and lower vertigo-specific functioning [VAP Scale 1: Beta = 2.47, 95%-CI (0.92; 4.02)], and VAP Scale 2: Beta = 3.74, 95%-CI [2.23; 5.24]).

**Conclusion:** Our results highlight the importance of a close monitoring of anticholinergic and sedative medication use in older patients with VDB. When feasible, anticholinergic and sedative medication should be replaced by equivalent alternative therapies in order to potentially reduce the burden of VDB.

## 1 Introduction

Vertigo, dizziness, and balance disorders (VDB) affect approximately 30% of the population beyond 60 years of age (Jönsson et al., 2004; Barin and Dodson, 2011), and up to 50% of those over 85 (Jönsson et al., 2004). A considerable percentage of older adults beyond 65 years with VDB experience severe impairment in their everyday life (van Vugt et al., 2020).

The reasons for VDB are often multifactorial. Distinct treatable vestibular disease entities, cardiovascular diseases or metabolic disorders may align with symptoms of the ageing of vestibular, proprioceptive or somatosensory systems.

In addition, VDB may be an unintended side effect of standard medication (Holt et al., 2010; Hedna et al., 2015; Muncie Jr et al., 2017). This is one of the reasons why a continuous medication review is recommended for older adults (Beuscart et al., 2021; Leitliniengruppe Hessen Deutsche Gesellschaft für Allgemeinmedizin und Familienmedizin, 2021). Medication that is causing VDB should be replaced by equivalent but better tolerated alternatives, whenever this is possible.

Of particular interest in this context is medication with anticholinergic and sedative (AS) effects. AS medication is prescribed for a multitude of indications, including urinary incontinence, sleep disturbances, mental illness, pain, cardiovascular diseases, and gastrointestinal disorders (Kouladjian et al., 2014). Certain anticholinergic medication (e.g., Scopolamine or Dimenhydrinate) and selected sedatives (e.g., Diazepam or Lorazepam) might also be prescribed as vestibular suppressants, i.e., drugs that reduce the subjective symptoms and intensity of vertigo as well as the nystagmus evoked by vestibular imbalance, in symptomatic therapy of VDB (Casani et al., 2021).

Previous research has shown that use of AS medication was higher in people with VDB than in the general population, especially in the old aged (Phillips et al., 2018; Phillips et al., 2019). This is of particular concern, since AS medication can lead to adverse effects such as confusion, blurred vision, delirium, and dizziness (Bell et al., 2012; Swain, 2020). Due to these adverse effects, a number of medication with AS properties such as selected antihistamines, urological spasmolytic agents, and benzodiazepines have been assessed as being potentially inappropriate for the general older population in Germany (Holt et al., 2010). The drug burden index (Hilmer et al., 2007) quantifies the combination of AS active substances with their respective dosage in a specific individual. Specifically in older adults, cognitive and physical impairment may be the consequence of a high AS drug burden (Landi et al., 2007; Cao et al., 2008; Hilmer et al., 2009; Koyama et al., 2014; Wouters et al., 2017; Byrne et al., 2019).

While the general association of AS medication and impaired functioning is well established, its specific role in individuals who are already affected by chronic or acute VDB is still incompletely understood.

It seems reasonable to hypothesize that use of AS medication considerably contributes to impaired functioning in two ways: First, it causes difficulties in performing activities of daily living (generic-functioning), such as hygiene, eating, grip, walking, and common daily activities. Furthermore, the use of AS medication could intensify the disease and therewith its direct impairment on the everyday life, especially on daily activities and social participation (vertigo-specific functioning).

The objective of this study thus was to investigate the impact of AS medication on both generic and vertigo-specific functioning in older adults with VDB.

## 2 Materials and methods

### 2.1 Study design, study population, and data collection procedures

Data for this research project was collected in the longitudinal multicenter study MobilE-TRA conducted in two German federal states (Bavaria and Saxony) from September 2017 until October 2019. A more detailed description of the study is given elsewhere (Kisch et al., 2018).

In short, patients aged 65 years and older were included if they had consulted their primary care physician (PCP) for an episode of VDB in the last quarter. The identification of suitable individuals was accomplished by approaching PCPs who were willing to participate and asking them to search their patient databases for the following ICD-10 codes associated with VDB: R42, A88.1, E53.8, F45.8, G11.8, G43.1, G45.0, G62, G63, H55, H83.0-2, I95.1, and N95.1. A detailed description of the diagnoses related to the ICD-10 codes is provided in the Supplementary Table S1. Additional inclusion criteria were a statutory health insurance, covering approximately 90% of the German population (The Federal Ministry of Health, 2020), as well as sufficient command of the German language.

Data collection consisted of three waves. The baseline assessment in between September 2017 and August 2018 comprised paper-based self-administered health questionnaires for both the patients and the PCPs. For the two follow-ups 6 months and 12 months after individual baseline dates, a cover letter with instructions and the paper-based self-administered health questionnaire were sent *via* postal mail to the patients’ home addresses. If no questionnaire was sent back within a month after the original follow-up invitation, a reminder was sent. In case of questions about the questionnaire, participants were able to contact the study team *via* e-mail or phone. There was no loss to follow-up between the study waves.

Ethics approval for MobiLE-TRA was obtained from the Ethics Committee of the Ludwig Maximilian University of Munich in Bavaria (#17-443) and the Ethics Committee of the Technical University Dresden in Saxony (#E365092017). Written informed consent was obtained from all participants. The study was performed in accordance with the Declaration of Helsinki principles.

### 2.2 Exposure to anticholinergic and sedative medication

Information on medication intake was obtained by the participants self-report during baseline assessment. Active pharmaceutical ingredients, the associated national identification number, and the prescribed daily dose for each medication taken within the last 7 days were recorded. The respective codes of the Anatomical Therapeutic Chemical Classification System (ATC-Codes) were matched to the national identification number.

AS medication was identified based on previous studies (Phillips et al., 2018; Phillips et al., 2019) using applicable published AS medication lists (Hilmer et al., 2007; Durán et al., 2013; Ailabouni et al., 2017; Wouters et al., 2017; Byrne et al., 2018; O'Connell et al., 2018) and potentially inappropriate AS medication from the German PRISCUS List (Holt et al., 2010), a collection of potentially inappropriate medication for older people. A detailed list of all AS medication and the related ATC-Codes present in this study sample is shown in Supplementary Table S2.

Since some AS medication might also be prescribed as vestibular suppressants in symptomatic therapy of VDB, we checked the information on prescribed drugs specifically for treatment of VDB, which was provided by the PCPs for each patient, in order to mitigate confounding by indication. No prescribed AS medication for treatment of VDB was detected.

To quantify the extent of the drug burden by AS medication at baseline, we calculated the drug burden index (DBI) (Hilmer et al., 2007). A DBI greater than zero represents a present AS drug burden with a higher index indicating higher AS burden.

The DBI of each participant was calculated using the following formula, where D denotes the prescribed daily dose of any AS medication for this participant, whereas *δ* is the defined daily dose (DDD) for this AS medication according to the Federal Institute for Drugs and Medical Devices (GKV-Arzneimittelindex im Wissenschaftlichen Institut der AOK, 2022):

$$DBI=\sum \frac{D}{\delta +D}$$

In order to ensure an adequate calculation of the DBI, we excluded all pro re nata medication as well as medication that was applied topically, ophthalmologically or by inhalation, since these dosing forms of medication have not been clearly defined (Kouladjian et al., 2014). Medication that was classified as having both anticholinergic and sedative effects was included only once in the calculation of the DBI (Hilmer et al., 2007; Best et al., 2013).

To handle the observed zero-inflation in the DBI of the study sample, exposure to AS medication at baseline was categorized following published cut-off points (Hilmer et al., 2007) into: No exposure (DBI = 0), low exposure (0 < DBI <1), and high exposure (DBI ≥1). Due to low observation numbers in the high exposure group, the DBI further was dichotomized into present (DBI greater than zero) and absent (DBI equals zero).

### 2.3 Generic and vertigo-specific functioning

Generic patient-reported functioning was assessed at baseline and both follow-ups by the German version of the Health Assessment Questionnaire Disability Index (HAQ-DI) (Fries et al., 1982; Brühlmann et al., 1994) in which patients reported the amount of difficulty they have in performing activities of daily living and instrumental activities of daily living (ADL and IADL). The overall HAQ-DI index was obtained by using the highest score within each of the eight domains (dressing and grooming, hygiene, arising, reach, eating, grip, walking, and common daily activities) and averaging these values into an overall HAQ-DI value (range = 0–3). Higher values in this overall index indicated stronger difficulties.

Vertigo-specific functioning was assessed at baseline and both follow-ups by the Vestibular Activities and Participation questionnaire (VAP) (Mueller et al., 2015). The VAP measures functioning in two dimensions by using two separate scales consisting of six items each. VAP Scale one measures patient-reported functioning regarding activities that are difficult to perform because of their propensity to provoke VDB (activity VAP). VAP Scale two indicates immediate consequences of VDB on activities and participation related to mobility (mobility VAP). Interval scaled overall scores (range scale 1 = 0–23; range scale 2 = 0–20) were used with higher scores indicating lower functioning.

### 2.4 Additional covariates

VDB diagnosis for each patient was reported by the respective PCP during baseline assessment as part of the self-administered health questionnaires given to the PCPs. Previous research has shown that unspecific diagnoses of VDB are remarkably over-diagnosed in primary care, resulting in a lack of adequate treatment for the actual underlying cause of VDB in the patients affected (Geser and Straumann, 2012). This lack of adequate treatment might manifest itself in reduced functioning. To account for this possibility and to facilitate analysis, we categorized the diagnoses with a specific cause (e.g., vestibular vertigo, central vertigo, cardiovascular problems, and psychogenic dizziness) as ‘specific’ VDB, whereas all other cases where no diagnostic decision was stated in the questionnaire were summarized as ‘unspecific’ VDB. The diagnosis was labeled as ‘not specified’ if the PCP did not specify any diagnosis in the questionnaire but enrolled the patient in the VDB survey.

Multimorbidity was included into the analysis since it increases the probability for medication and thereby the risk of unwanted effects in older people (Vrdoljak and Borovac, 2015) and is associated with reduced functioning (Loza et al., 2009). Multimorbidity was present, if a patient suffered from at least two chronic conditions additionally to VDB during baseline assessment. The assessment of chronic conditions in Mobile-TRA was accomplished by asking the PCP for existing comorbidities during baseline assessment and was based on the self-report-generated Charlson Comorbidity Index (Chaudhry et al., 2005), excluding HIV. Following (Kirchberger et al., 2012), further comorbidities that had shown to be of high relevance when examining multimorbidity with increasing age (Hunger et al., 2011) were added. Our approach results in an overall of 12 chronic conditions (Supplementary Material S1).

Information on gender (male/female) and age was based on patients’ self-report. Federal states were included as a binary variable (Bavaria/Saxony).

### 2.5 Statistical analysis

Unadjusted summary statistics were calculated for the overall sample and separately for patients with and patients without exposure to AS medication to compare differences at baseline regarding diagnoses and characteristics of the patients as well as generic and vertigo-specific functioning. Median values and the interquartile range (IQR) between the 25% and 75% - quartiles were reported for continuous variables, relative and absolute frequencies were reported for categorical variables. Group comparison between patients with and patients without exposure to AS medication was based on Kruskal-Wallis-Test for continuous variables and Fisher’s exact test for categorical variables.

We applied longitudinal linear mixed models with random intercepts and fixed slopes to assess the association of exposure to AS medication at baseline, represented by the dichotomized DBI, and the level of generic (HAQ-DI) and vertigo-specific (VAP) functioning status over time. To analyze the effect of baseline exposure on the change in functioning over time, we introduced an interaction term between AS exposure status at baseline and time. Multicollinearity among predictor variables was tested by calculating the variance inflation factor (VIF) for each model, with a highest tolerated VIF lower than five points.

In order to investigate the effect of the level of the AS drug burden, we additionally performed sensitivity analyses, computing the same longitudinal linear mixed models based on the categorized DBI values.

Random effects for the intercepts were reported. Intraclass correlation coefficients (ICC) were introduced for each model to examine the proportion of the overall variation in the respective functioning scale which is explained by the AS medication exposure status at baseline. To facilitate the interpretation of intercept estimates, we subtracted the minimum age of 65 as set by the inclusion criteria from the age in years for every patient in all mixed models.

All computational analyses were carried out with R version 4.1.0 (RStudio Team, 2020) using the nlme and misty libraries (Pinheiro et al., 2007; Yanagida and Yanagida, 2022). Significance level was set to 5% for all tests conducted.

## 3 Results

### 3.1 Study population

An overall of 19 PCPs (7 from Bavaria, 12 from Saxony; mean age = 54 years; 29% female) recruited 158 patients with VDB (59% from Bavaria; mean age = 77 years; 70% female). Exposure to AS medication at baseline was present in 56 (35%) patients. Of these, 49 (31%) patients had a low AS exposure status (0 < DBI<1), whereas 7 (4%) patients had a high AS exposure status (DBI ≥1). A total of 42% of the patients had a specific VDB diagnosis, 40% of the patients had an unspecific VDB diagnosis, and the VDB diagnosis was not specified in 18% of the patients. Median HAQ-DI at baseline was 0.38, median activities VAP was 7.36, and mean mobility VAP was 6.42. Patients exposed to AS medication reported significantly higher values for the HAQ-DI and both scales of the VAP scales during baseline assessment. Further details are presented in Table 1.

**TABLE 1:** Unadjusted summary statistics by exposure to AS medication at baseline assessment (n = 158). Median values and interquartile range for continuous variables and absolute and relative frequencies for categorical variables are reported.

		Exposure to AS medication			
	Overall	No (DBI = 0)	Low (0 < DBI < 1)	High (DBI ≥ 1)	*p*-value
N (%)	158 (100)	102 (65)	49 (31)	7 (4)	
Diagnosis of VDB					
Specific (n., %)	67 (100)	42 (63)	24 (36)	1 (1)	0.524
Unspecific (n., %)	29 (100)	19 (66)	8 (27)	2 (7)	
Not specified (n., %)	62 (100)	41 (66)	17 (27)	4 (7)	
Multimorbidity^a^					
No (n., %)	30 (100)	18 (60)	11 (37)	1 (3)	0.740
Yes^a^ (n., %)	128 (100)	84 (66)	38 (30)	6 (4)	
Gender					
Male (n., %)	48 (100)	30 (62)	17 (36)	1 (2)	0.513
Female (n., %)	110 (100)	72 (65)	32 (29)	6 (6)	
Age (median, IQR)	78.00 (72.00; 82.00)	78.00 (73.00; 82.00)	79.00 (72.00; 82.00)	76.00 (73.00; 80.00)	0.922
Study location					
Bavaria (n., %)	94 (100)	62 (66)	27 (29)	5 (5)	0.645
Saxony (n., %)	64 (100)	40 (63)	22 (34)	2 (3)	
Generic functioning					
HAQ-DI (median, IQR)	0.38 (0.01; 1.00)	0.25 (0.00; 0.75)	0.62 (0.12; 1.38)	1.12 (0.75; 1.44)	0.002
Vertigo-specific Functioning					
Activity VAP Scale1 (median, IQR)	7.36 (4.38; 10.57)	6.55 (3.04; 9.82)	9.67 (5.83; 12.06)	11.76 (9.34; 13.79)	0.006
Mobility VAP Scale 2 (median, IQR)	6.42 (3.75; 9.61)	5.69 (2.21; 8.33)	7.71 (4.83; 12.60)	13.15 (10.51; 5.56)	0.001

AS, anticholinergic and sedative; DBI, drug burden index; VDB, vertigo, dizziness, and balance disorders; HAQ-DI, health assessment questionnaire disability index; VAP, vestibular activities and participation questionnaire; SD, standard deviation; IQR, interquartile range.

^a^ ‘Yes’, if patient suffered from at least two chronic conditions in addition to VDB, during baseline assessment.

*p*-values based on Kruskal-Wallis-Test for continuous variables and Fisher’s exact test for categorical variables.

### 3.2 Impact of AS medication on generic and vertigo-specific functioning

Table 2 shows the adjusted estimates for the association of exposure to AS medication at baseline and generic and vertigo-specific functioning. Adjusted for covariates, generic functioning was significantly lower for patients that were exposed to AS medication at baseline [Beta = 0.40, 95%-CI (0.18; 0.61)]. Exposure to AS medication at baseline accounted for nine percent of the total variance observed in the generic functioning (ICC_HAQ-DI = 0.09). Exposure to AS medication at baseline also was significantly associated with higher values on the activity VAP Scale 1 [Beta = 2.47, 95%-CI (0.92; 4.02)] and mobility VAP Scale 2 [Beta = 3.74, 95%-CI (2.23; 5.24)], indicating lower vertigo-specific functioning. Exposure to AS medication at baseline accounted for twelve percent of the total variance observed in the activity VAP Scale 1 (ICC_VAP1 = 0.12) and 17 percent of the total variance observed in the mobility VAP Scale 2 (ICC_VAP2 = 0.17). Impairment in vertigo-specific activity-related functioning (activity VAP Scale 1) declined over time for the overall sample [Beta = −0.47, 95%-CI (-0.91; −0.03)]. No significant differences in the development of functioning over time between patients that were exposed to AS medication at baseline and patients that were not exposed were detected.

**TABLE 2:** Longitudinal linear mixed models to assess the influence of exposure to AS medication on generic functioning (HAQ-DI) and vertigo-specific functioning (Activity VAP Scale one and Mobility VAP Scale 2).

	Generic functioning	Vertigo- specific functioning	
	HAQ-DI (95% CI)	Activity VAP Scale 1 (95% CI)	Mobility VAP Scale 2 (95% CI)
Observations (n)	435 (158)	273 (127)	310 (139)
**Fixed effects**			
Intercept	−0.27 (-0.67; 0.12)	1.59 (−1.01; 4.19)	−1.14 (−3.74; 1.47)
Wave	0.03 (−0.01; 0.07)	−**0.47 (**−**0.91;** −**0.03)**	0.04 (−0.38; 0.45)
Exposure to AS medication^a^ at baseline			
No	Reference	Reference	Reference
Yes	**0.40 (0.18; 0.61)**	**2.47 (0.92; 4.02)**	**3.74 (2.23; 5.24)**
Interaction terms exposure to AS medication at baseline * wave			
No * wave	Reference	Reference	Reference
Yes * wave	−0.01 (−0.07; 0.06)	0.27 (−0.49; 1.03)	−0.38 (−1.08; 0.31)
Diagnosis of VDB			
Specific	Reference	Reference	Reference
Unspecific	**0.27 (0.04; 0.50)**	1.28 (−0.27; 2.83)	**2.53 (1.04; 4.03)**
Not specified	0.26 (−0.03; 0.54)	**2.04 (0.16; 3.92)**	**2.38 (0.53; 4.24)**
Age^b^	**0.04 (0.02; 0.05)**	**0.16 (0.05; 0.27)**	**0.28 (0.17; 0.39)**
Study location			
Bavaria	Reference	Reference	Reference
Saxony	−0.05 (−0.27; 0.16)	0.24 (−1.22; 1.71)	0.73 (−0.69; 2.15)
Gender			
Male	Reference	Reference	Reference
Female	**0.30 (0.07; 0.53)**	**2.26 (0.69; 3.83)**	**2.55 (1.06; 4.05)**
Multimorbidity^c^			
No	Reference	Reference	Reference
Yes	−0.02 (−0.28; 0.25)	0.89 (−0.79; 2.57)	−0.24 (−1.90; 1.43)
**Random effects**			
Intercept (SD)	0.61	3.40	3.50
**ICC**	0.09	0.12	0.17

Significant results are highlighted in bold print.

AS, anticholinergic and sedative; VDB, vertigo, dizziness, and balance disorders; HAQ-DI, health assessment questionnaire disability index; VAP, vestibular activities and participation questionnaire; CI, confidence interval; ICC, Intraclass correlation coefficient.

^a^ ’Yes’, if drug burden index greater than zero.

^b^ The minimum age of 65 as set by the inclusion criteria was subtracted from age in years for each patient.

^c^ ‘Yes’, if patient suffered from at least two chronic conditions in addition to VDB, during baseline assessment.

Patients with an unspecific diagnosis had lower generic functioning [Beta = 0.27, 95%-CI (0.04; 0.50)] and lower vertigo-specific mobility [Beta = 2.53, 95%-CI (1.04; 4.03)]. Patients in which the VDB diagnosis remained unspecified reported significantly worse vertigo-specific functioning on both VAP scales. Older participants and women were significantly more disabled.

The performed sensitivity analyses based on the categorized DBI showed, that patients with a low exposure to AS medication (0 < DBI<1) showed lower generic functioning and lower vertigo-specific functioning, when compared to patients without any AS exposure (DBI = 0). Patients with a high exposure to AS medication (DBI ≥1) had even lower vertigo-specific functioning on the VAP Scale 1 [Beta = 3.45, 95%-CI (0.15; 6.75)] and mobility VAP Scale 2 [Beta = 5.63, 95%-CI (1.93; 9.32)]. Further details are presented in Supplementary Table S3.

## 4 Discussion

Using data from a German primary care-based longitudinal multicenter study, we have shown that an exposure to AS medication was associated with lower values in both generic and vertigo-specific functioning in older patients with VDB. Patients in which the underlying mechanism of VDB remained unspecified were particularly at risk of impaired functioning.

In our study, patients that were exposed to AS medication at baseline had lower generic functioning than patients who were not exposed to AS medication. This is in line with previous findings stating that use of AS medication is associated with functional impairments (Landi et al., 2007; Cao et al., 2008; Hilmer et al., 2009; Koyama et al., 2014; Wouters et al., 2017; Byrne et al., 2019), due to its adverse effects (Holt et al., 2010; Bell et al., 2012) that are increasing the amount of difficulty that patients have in performing activities of their daily living.

The most striking observation of our study is that lower vertigo-specific functioning also was associated with exposure to AS medication. Apart from increasing difficulties in performing general tasks of daily living, the use of AS medication also intensifies the direct major impairment on the everyday life, especially on daily activities, social participation, and mobility. To our knowledge, no study has yet explicitly investigated this before. These results are highly alerting since it has been noted that use of AS medication was higher in people with VDB than in the general population, especially in the old aged (Phillips et al., 2018; Phillips et al., 2019).

Regardless of their exposure to AS medication, patients diagnosed with unspecific VDB, i.e., cases in which the cause of VDB remained unspecified by the respective PCP, and patients in which the entire diagnosis of VDB remained unspecified by their PCP, had lower functioning than patients with a specific diagnosis. It has been mentioned that VDB, especially in older patients, can have multiple causes (Maarsingh et al., 2010; Fernández et al., 2015) and that the symptoms often are ambiguous. It therefore is of little surprise that unspecific VDB is frequently over-diagnosed in primary care by as much as up to 60%, when compare to diagnostic procedures at specialized care centers, possibly resulting in inadequate treatment (Geser and Straumann, 2012). This inadequate treatment might ultimately manifest itself in worse functioning.

Our findings demonstrate that women are at risk of lower generic and vertigo-specific functioning. This is in line with recent findings that women at the age of 65 and above in Germany expect to spend less of their remaining life years in good health than men, due to their higher morbidity and despite their higher life expectancy (Stephan et al., 2021). It further has been shown that the overall potentially inappropriate medication use was higher in older women (Nothelle et al., 2017; Nothelle et al., 2019), possibly contributing to the lower functioning found in this study.

Several limitations of our study have to be considered. Information on medication intake was based on self-report. Chances are that patients did not indicate a comprehensive list or that the indicated doses taken was inaccurate. The true extent of the exposure to AS medication thus might have been underestimated. Likewise, the assessment of generic functioning *via* the HAQ-DI and vertigo-specific functioning *via* the VAP was based on the participants’ self-report and thus might have potentially exposed to a variety of information bias. Though we cannot fully exclude the chance that such bias might be present in our data, self-reported measure is commonly seen as a valid outcome, assessing the patients’ perspective. Both the HAQ-DI and the VAP are standardized and validated instruments which have frequently used in the past. Patients further were offered contact information of the study team in case of any uncertainty regarding their study participation or the questionnaire in order to reduce the risk of bias. The DBI within this study was calculated using the DDD instead of the minimum effective dose as specified in the original calculation of the DBI (Hilmer et al., 2007) since the German ATC classification system was closely linked to the DDD. Using the DDD as a replacement of the minimum effective dose has been introduced in the past (Faure et al., 2013). This however might have resulted in an underestimation of the actual DBI, since the DBI is higher for some drugs, but not for others (Hilmer, 2018). Suitable subjects for this study were identified by searching for ICD-10 codes associated with VDB in the databases of the participating PCPs. The reliability of ICD-10 codes as a reliable classification system in primary care has been discussed in the past (Wockenfuss et al., 2009), especially since they became one of the corner stones of reimbursement in the German healthcare system and thus might be divergent from the actual diagnosis. While the diagnosis of VDB use in this analysis was given directly by the PCP as part of the questionnaire and therefore decoupled from the ICD codes, some potential participants might have been left out due to inaccurate ICD-10 codes that were not listed in our inclusion criteria. The VDB diagnoses used in this analysis were solely based on the assessments of the participating PCPs at baseline. This is of relevance, since previous research has shown that PCPs tend to frequently over-diagnose unspecific VDB in patients who later were diagnosed with a specific cause of VDB (Geser and Straumann, 2012) due to reported difficulties in establishing an accurate VDB diagnosis in the past (Stephan et al., 2018). Also, we do not know if (and when) the PCP or specialist made a specific diagnosis during the follow-up time. While the list of assessed comorbidities within this study was comprehensive, it did not include all comorbidities that are associated with the use of AS medication, such as sleep and pain disorders, urinary incontinence, mental disorders, and dementia (Kouladjian et al., 2014). Single comorbidities might also differ with regard to their relation with VDB, the prescription frequency of AS medication, and their direct impact on generic and vertigo-specific functioning. Using multimorbidity as a substitute, the estimation of the impact of AS medication on functioning might be distorted due to unaccounted confounders. We therefore strongly suggest to further review our findings, including a more detailed assessment of the present medication intake and existing comorbidities.

In conclusion, we found that an exposure to AS medication was associated with lower values in both generic and vertigo-specific functioning in older patients with VDB. When feasible, AS medication thus should be replaced by equivalent alternative therapies that are adapted to the situation and needs of each patient individually. Valid approaches have already been examined and include change of medication as well as non-pharmacological treatment, such as physical exercises or behavioral therapy (Holt et al., 2010). The use of AS medication in symptomatic therapy of VDB remains debated (Hunter et al., 2022). Our results support recent recommendations that their use as vestibular suppressants should be limited only to the acute phase of the disease and must be used with special caution in the older population (Casani et al., 2021).

A close monitoring of AS medication use in older patients with VDB symptoms is crucial and should be considered as an integral component in medication monitoring guidelines in a primary care setting, as, for example, happened in the German DEGAM-Guideline (S3) for polypharmacy (Leitliniengruppe Hessen Deutsche Gesellschaft für Allgemeinmedizin und Familienmedizin (DEGAM), 2021). Using the drug burden index could be a valid approach to do so and should be accompanied by implementation studies in order to ensure feasibility. In doing so, exposure to AS medication could be reduced and thus help to potentially reduce the burden of VDB.

Previous research aimed at evaluating the utility of the DBI in practice showed that, while using the DBI as an assessment tool in older adults can have the potential to reduce AS burden in some studies (Nishtala et al., 2009; Castelino et al., 2010), the effect could not always be shown (van der Meer et al., 2018) or was lower than anticipated (Gnjidic et al., 2010). Agreement is that future research needs to be implemented targeting multidisciplinary and multifactorial approaches, including the DBI, to evaluate drug prescription and to evaluate functional outcomes in older adults (Kouladjian et al., 2014).

## Data Availability

The datasets presented in this article are not readily available because of data privacy regulations that apply in the country of data collection. Requests to access the datasets should be directed to the corresponding author BK (benedict.katzenberger@med.uni-muenchen.de).
