# Supplementary material for: Exposure to anticholinergic and sedative medication is associated with impaired functioning in older people with vertigo, dizziness and balance disorders—Results from the longitudinal multicenter study MobilE-TRA
Source: Front Pharmacol. 2023 Mar 3;14:1136757. doi: 10.3389/fphar.2023.1136757 (PMC10020174; doi:10.3389/fphar.2023.1136757)
Supplement: Supplementary file 1 [file Table1.docx]

Supplementary Material

**Exposure to anticholinergic and sedative medication is associated with impaired functioning in older people with vertigo, dizziness and balance disorders – Results from the longitudinal multicenter study MobilE-TRA**

**Benedict Katzenberger*, Daniela Koller, Ralf Strobl, Rebecca Kisch, Linda Sanftenberg, Karen Voigt, Eva Grill**

*** Correspondence:** Benedict Katzenberger: Benedict.Katzenberger@med.uni-muenchen.de

**Supplementary Table S1. ICD-10 codes associated with vertigo, dizziness, and balance disorders and related diagnoses in the study MobilE-TRA**

| **ICD-10 code** | **VDB diagnosis** |
| --- | --- |
| R42 | Dizziness and giddiness |
| A88.1 | Epidemic vertigo |
| E53.8 | Deficiency of other specified B group vitamins |
| F45.8 | Other somatoform disorders |
| G11.8 | Other hereditary ataxias |
| G43.1 | Other headache syndromes |
| G45.0 | Vertebro-basilar artery syndrome |
| G62 | Other polyneuropathies |
| G63 | Polyneuropathy in diseases classified elsewhere |
| H55 | Nystagmus and other irregular eye movements |
| H83.0–2 | Other diseases of inner ear |
| I95.1 | Orthostatic hypotension |
| N95.1 | Menopausal and female climacteric states |

ICD-10, International classification of diseases, 10th Revision; VDB = Vertigo, dizziness and balance disorders
