# Supplementary material for: Exposure to anticholinergic and sedative medication is associated with impaired functioning in older people with vertigo, dizziness and balance disorders—Results from the longitudinal multicenter study MobilE-TRA
Source: Front Pharmacol. 2023 Mar 3;14:1136757. doi: 10.3389/fphar.2023.1136757 (PMC10020174; doi:10.3389/fphar.2023.1136757)
Supplement: Supplementary file 2 [file Table2.docx]

Supplementary Material

**Exposure to anticholinergic and sedative medication is associated with impaired functioning in older people with vertigo, dizziness and balance disorders – Results from the longitudinal multicenter study MobilE-TRA**

**Benedict Katzenberger*, Daniela Koller, Ralf Strobl, Rebecca Kisch, Linda Sanftenberg, Karen Voigt, Eva Grill**

*** Correspondence:** Benedict Katzenberger: Benedict.Katzenberger@med.uni-muenchen.de

**Supplementary Table S2. Medications and ATC-Codes of AS medication present in the study**

| Drug | WHO ATC Code/s | Anticholinergic Effects (AC) | Sedative Effects (S) |
| --- | --- | --- | --- |
| Acetyldigoxin | C01AA02 | AC |  |
| Alfuzosin | G04CA01 |  | S |
| Alprazolam | N05BA12 |  | S |
| Amitriptyline | N06AA09 | AC | S |
| Bromazepam | N05BA08 |  | S |
| Brotizolam | N05CD09 |  | S |
| Carbamazepine | N03AF01 | AC | S |
| Cetirizine | R06AE07 | AC |  |
| Citalopram | N06AB04 | AC | S |
| Clonazepam | N03AE01 | AC | S |
| Darifenacin | G04BD10 | AC |  |
| Diazepam | N05BA01 |  | S |
| Diclofenac | M01AB05 | AC |  |
| Digitoxine | C01AA04  C01AA05 | AC |  |
| Dimenhydrinat | A04AB02 | AC |  |
| Domperidone | A03FA03 | AC |  |
| Doxazosin | C02CA04 |  | S |
| Doxepin | N06AA12 | AC | S |
| Duloxetine | N06AX21 | AC | S |
| Ebastin | R06AX22 | AC |  |
| Escitalopram | N06AB10 | AC | S |

*continued on next page*

*Supplementary Table S2. continued*

| Drug | WHO ATC Code/s | Anticholinergic Effects (AC) | Sedative Effects (S) |
| --- | --- | --- | --- |
| Fentanyl | N02AB03 | AC | S |
| Hydromorphone | N02AA03 | AC | S |
| Loperamid | A07DA03 | AC |  |
| Loratadine | R06AX13 | AC |  |
| Lorazepam | N05BA06 |  | S |
| Metoclopramide | A03FA01 | AC |  |
| Mirabegron | G04BD12 | AC |  |
| Mirtazepine | N06AX11 | AC | S |
| Morphine | N02AA01 |  | S |
| Moxonidine | C02AC05 |  | S |
| Opipramol | N06AA05 | AC | S |
| Oxycodone | N02AA05 | AC | S |
| Oxycodone and Naloxone | N02AA55  N02AA56 | AC | S |
| Pramipexole | N04BC05 | AC |  |
| Quetiapine | N05AH04 | AC | S |
| Rotigotine | N04BC09 | AC |  |
| Sertraline | N06AB06 | AC | S |
| Solifenacin | G04BD08 | AC |  |
| Tamsulosin | G04CA02 |  | S |
| Tapentadol | N02AX06 | AC | S |
| Theophylline | R03DA04 | AC |  |
| Tilidine | N02AX01 | AC | S |
| Tilidine and Naloxone | N02AX51 | AC | S |
| Tolterodine | G04BD07 | AC |  |
| Tolperison | M03BX04 | AC |  |
| Tramadol | N02AX02 | AC | S |
| Trimipramine | N06AA06 | AC | S |
| Trospium | G04BD09 | AC |  |
| Urapidil | C02CA06 |  | S |
| Valerian root | N05CP01 |  | S |
| Venlafaxine | N06AX16 | AC | S |
| Zolpidem | N05CF02 |  | S |
| Zopiclone | N05CF01 |  | S |
