# Supplementary material for: Exposure to anticholinergic and sedative medication is associated with impaired functioning in older people with vertigo, dizziness and balance disorders—Results from the longitudinal multicenter study MobilE-TRA
Source: Front Pharmacol. 2023 Mar 3;14:1136757. doi: 10.3389/fphar.2023.1136757 (PMC10020174; doi:10.3389/fphar.2023.1136757)
Supplement: Supplementary file 3 [file Table3.docx]

Supplementary Material

**Exposure to anticholinergic and sedative medication is associated with impaired functioning in older people with vertigo, dizziness and balance disorders – Results from the longitudinal multicenter study MobilE-TRA**

**Benedict Katzenberger*, Daniela Koller, Ralf Strobl, Rebecca Kisch, Linda Sanftenberg, Karen Voigt, Eva Grill**

*** Correspondence:** Benedict Katzenberger: Benedict.Katzenberger@med.uni-muenchen.de

**Supplementary Table S3. Longitudinal linear mixed models to assess the effect of the level of the AS drug burden on generic functioning (HAQ-DI) and vertigo-specific functioning (activity VAP Scale 1 and mobility VAP Scale 2).**

|  | | Generic functioning |  | Vertigo- specific functioning | |
| --- | --- | --- | --- | --- | --- |
|  | | HAQ-DI  (95% CI) |  | Activity VAP Scale 1  (95% CI) | Mobility VAP Scale 2  (95% CI) |
| Observations (n) | | 435 (158) |  | 273 (127) | 310 (139) |
|  | |  |  |  |  |
| **Fixed effects** | |  |  |  |  |
| Intercept | | -0.27 (-0.67 ; 0.12) |  | 1.68 (-0.92 ; 4.29) | -1.03 (-3.62 ; 1.57) |
| Wave | | 0.03 (-0.01 ; 0.07) |  | **-0.47 (-0.91 ; -0.03)** | 0.04 (-0.38 ; 0.45) |
| Exposure to AS medication at baseline | |  |  |  |  |
|  | No (DBI = 0) | Reference |  | Reference | Reference |
|  | Low (0<DBI<1) | **0.39 (0.17 ; 0.62)** |  | **2.28 (0.64 ; 3.93)** | **3.45 (1.87 ; 5.02)** |
|  | High (DBI ≥1) | 0.45 (-0.06 ; 0.95) |  | **3.45 (0.15 ; 6.75)** | **5.63 (1.93 ; 9.32)** |
| Interaction terms exposure to AS medication at baseline * wave | |  |  |  |  |
|  | No * wave | Reference |  | Reference | Reference |
|  | Low (0<DBI<1) * wave | -0.01 (-0.08 ; 0.06) |  | 0.27 (-0.52 ; 1.06) | -0.37 (-1.07 ; 0.34) |
|  | High (DBI ≥1) * wave | 0.01 (-0.15 ; 0.18) |  | 0.37 (-1.61 ; 2.36) | -0.40 (-2.67 ; 1.87) |
| Diagnosis of VDB | |  |  |  |  |
|  | Specific | Reference |  | Reference | Reference |
|  | Unspecific | **0.26 (0.03 ; 0.50)** |  | 1.21 (-0.34 ; 2.77) | **2.40 (0.90 ; 3.90)** |
|  | Not specified | 0.25 (-0.03 ; 0.54) |  | **1.96 (0.08 ; 3.85)** | **2.34 (0.49 ; 4.18)** |
| Age^a^ | | **0.04 (0.02 ; 0.05)** |  | **0.16 (0.06 ; 0.27)** | **0.28 (0.17 ; 0.39)** |
| Study location | |  |  |  |  |
|  | Bavaria | Reference |  | Reference | Reference |
|  | Saxony | -0.05 (-0.27 ; 0.17) |  | 0.26 (-1.20 ; 1.72) | 0.79 (-0.62 ; 2.21) |
| Gender | |  |  |  |  |
|  | Male | Reference |  | Reference | Reference |
|  | Female | **0.30 (0.07 ; 0.52)** |  | **2.21 (0.63 ; 3.78)** | **2.49 (1.00 ; 3.98)** |
| Multimorbidity^b^ | |  |  |  |  |
|  | No | Reference |  | Reference | Reference |
|  | Yes | -0.02 (-0.28 ; 0.24) |  | 0.84 (-0.84 ; 2.52) | -0.26 (-1.92 ; 1.39) |
|  |  |  |  |  |  |
| **Random effects** | |  |  |  |  |
| Intercept (SD) | | 0.61 |  | 3.39 | 3.47 |
| **ICC** | | 0.11 |  | 0.18 | 0.30 |

*Significant results are highlighted in bold print.
AS = anticholinergic and sedative; DBI = Drug Burden Index; VDB = Vertigo, dizziness, and balance disorders;
HAQ-DI = Health Assessment Questionnaire Disability Index; VAP = Vestibular Activities and Participation questionnaire; CI = Confidence interval; ICC = Intraclass correlation coefficient.
^a^The minimum age of 65 as set by the inclusion criteria was subtracted from age in years for each patient;
^b^’Yes’, if patient suffered from at least two chronic conditions in addition to VDB during baseline assessment.*
