# Supplementary material for: Exposure to anticholinergic and sedative medication is associated with impaired functioning in older people with vertigo, dizziness and balance disorders—Results from the longitudinal multicenter study MobilE-TRA
Source: Front Pharmacol. 2023 Mar 3;14:1136757. doi: 10.3389/fphar.2023.1136757 (PMC10020174; doi:10.3389/fphar.2023.1136757)
Supplement: Supplementary file 4 [file DataSheet1.docx]

Supplementary Material

Exposure to anticholinergic and sedative medication is associated with impaired functioning in older people with vertigo, dizziness and balance disorders – Results from the longitudinal multicenter study MobilE-TRA

**Benedict Katzenberger*, Daniela Koller, Ralf Strobl, Rebecca Kisch, Linda Sanftenberg, Karen Voigt, Eva Grill**

*** Correspondence:** Benedict Katzenberger: Benedict.Katzenberger@med.uni-muenchen.de

**Supplementary Material S1. Assessment of comorbidities in the study MobilE-TRA**

The assessment of the comorbidities was accomplished by asking the patient’s primary care physician (PCP) about the present comorbidities during the baseline assessment. Comorbidities were reported by the PCP using the Charlson Comorbidity Index (Chaudhry et al., 2005). Following recommendations (Kirchberger et al., 2012), we added further comorbidities to the index list that had shown to be of high relevance in older adult populations. The PCPs were asked about the presence of the following comorbidities:

- pulmonary diseases, such as asthma, emphysema, and chronic bronchitis
- inflammatory joint disease (e.g. arthritis) or rheumatism
- cancer and other malignant tumor diseases
- diabetes mellitus
- gastrointestinal disease (e.g. stomach or duodenal ulcer, colon inflammation, cholecystitis)
- heart disease (e.g. angina pectoris, cardiac insufficiency, coronary heart disease)
- stroke
- other neurologic disease (e.g. multiple sclerosis, Parkinson’s disease, epilepsy)
- kidney disease
- liver disease (e.g. cirrhosis)
- eye diseases (excluding hyperopia and myopia)
- hypertension

**References**

Chaudhry, S., Jin, L., and Meltzer, D. (2005). Use of a self-report-generated Charlson Comorbidity Index for predicting mortality. *Med Care***,** 607-615. doi: 10.1097/01.mlr.0000163658.65008.ec.

Kirchberger, I., Meisinger, C., Heier, M., Zimmermann, A.-K., Thorand, B., Autenrieth, C.S., et al. (2012). Patterns of multimorbidity in the aged population. Results from the KORA-Age study. *PloS One* 7(1)**,** e30556. doi: 10.1371/journal.pone.0030556.
